# Internal breast dosimetry in mammography: Monte Carlo validation in homogeneous and anthropomorphic breast phantoms with a clinical mammography system

**DOI:** 10.1002/mp.13069

**Published:** 2018-07-18

**Authors:** Christian Fedon, Marco Caballo, Ioannis Sechopoulos

**Affiliations:** ^1^ Department of Radiology and Nuclear Medicine Radboud University Medical Center PO Box 9101 6500 HB Nijmegen The Netherlands; ^2^ Dutch Expert Center for Screening (LRCB) PO Box 6873 6503 GJ Nijmegen The Netherlands

**Keywords:** 3D‐breast phantom, breast dosimetry, GEANT4, experimental measurements, dose distribution

## Abstract

**Purpose:**

To validate Monte Carlo (MC)‐based breast dosimetry estimations using both a homogeneous and a 3D anthropomorphic breast phantom under polyenergetic irradiation for internal breast dosimetry purposes.

**Methods:**

Experimental measurements were performed with a clinical digital mammography system (Mammomat Inspiration, Siemens Healthcare), using the x‐ray spectrum selected by the automatic exposure control and a tube current‐exposure time product of 360 mAs. A homogeneous 50% glandular breast phantom and a 3D anthropomorphic breast phantom were used to investigate the dose at different depths (range 0–4 cm with 1 cm steps) for the homogeneous case and at a depth of 2.25 cm for the anthropomorphic case. Local dose deposition was measured using thermoluminescent dosimeters (TLD), metal oxide semiconductor field‐effect transistor dosimeters (MOSFET), and GafChromic™ films. A Geant4‐based MC simulation was modified to match the clinical experimental setup. Thirty sensitive volumes (3.2 × 3.2 × 0.38 mm^3^) on the axial‐phantom plane were included at each depth in the simulation to characterize the internal dose variation and compare it to the experimental TLD and MOSFET measurements. The experimental 2D dose maps obtained with the GafChromic™ films were compared to the simulated 2D dose distributions.

**Results:**

Due to the energy dependence of the dosimeters and due to x‐ray beam hardening, dosimeters based on these three technologies have to be calibrated at each depth of the phantom. As expected, the dose was found to decrease with increasing phantom depth, with the reduction being ~93% after 4 cm for the homogeneous breast phantom. The 2D dose map showed nonuniformities in the dose distribution in the axial plane of the phantom. The mean combined standard uncertainty increased with phantom depth by up to 5.3% for TLD, 6.3% for MOSFET, and 9.6% for GafChromic™ film. In the case of a heterogeneous phantom, the dosimeters are able to detect local dose gradient variations. In particular, GafChromic™ film showed local dose variations of about 46% at the boundaries of two materials.

**Conclusions:**

Results showed a good agreement between experimental measurements (with TLD and MOSFET*)* and MC data for both homogeneous and anthropomorphic breast phantoms. Larger discrepancies are found when comparing the GafChromic™ dose values to the MC results due to the inherent less precise nature of the former.

MC validations in a heterogeneous background at the level of local dose deposition and in absolute terms play a fundamental role in the development of an accurate method to estimate radiation dose. The potential introduction of a breast dosimetry model involving a nonhomogeneous glandular/adipose tissue composition makes the validation of internal dose distributions estimates crucial.

## 1 Introduction

Currently, digital mammography is used as the primary diagnostic technology for early detection of breast cancer. For this x‐ray based imaging modality, the estimation of the absorbed dose to the breast is part of quality control procedures.^1^ Due to the exposure to x rays, there is a risk of carcinogenesis in all mammography examinations. This risk, albeit small,^2^ has to be understood. Thus, an accurate and controlled evaluation of the delivered dose is important.

The radiation dose metric for mammography has developed considerably since the late 1970s.^3^ The mean glandular dose (MGD) is the currently accepted dosimetric quantity for breast dose evaluation. This quantity acknowledges that the fibroglandular tissue is the most radiosensitive component of the breast. Current models involve a few simplifying assumptions regarding breast shape and internal composition.^4^, ^5^, ^6^, ^7^

Concerning the breast shape, current models only represent the breast using a semi‐elliptical approximation of the cranio‐caudal (CC) view,^8^, ^9^ while only a subjective model was proposed for the medio‐lateral oblique (MLO) view.^10^ Objective analysis of the compressed breast undergoing mammography has resulted in new models for both the CC and MLO views.^11^, ^12^ Recently, the 3D curvature of the compressed breast between the support table and the compression paddle has been also characterized.^13^

In addition, the breast models assume that the fibroglandular tissue is uniformly distributed within a defined breast region and is perfectly mixed with the adipose tissue. Models with varying glandular percentage between pure adipose (i.e., 0% glandular tissue) and pure glandular (i.e., 100% glandular tissue) can be defined. This homogeneous tissue approximation introduces an overestimation in the dose evaluations as shown by the works of Dance et al.,^14^ Sechopoulos et al.,^15^ and Hernandez et al.^16^

These studies pointed out the importance in investigating the effect of assuming a simple homogeneous distribution against a heterogeneous distribution of the glandular tissue in the breast. Due to the availability of 3D breast images obtained by breast computed tomography, a model of the real 3D glandular tissue in the breast is feasible,^17^ leading to the possibility of an improved dose estimate. In addition, the acquisition of 3D glandular tissue distribution information from breast tomosynthesis, albeit limited, could allow for patient‐specific dose estimates.

For this task, Monte Carlo (MC) computer simulations play a crucial role in dose estimation, due to the fact that a direct measurement of MGD is not feasible. Hence, conversion factors from incident air kerma to MGD, obtainable only with MC simulations, must be used. Therefore, MC simulations need to be validated before their results can be considered reliable.

In our previous work,^18^ we proposed experimental methodologies to validate MC simulations using three different dosimeters [GafChromic™ films, thermoluminescent dosimeters (TLDs), and metal oxide semiconductor field‐effect transistor (MOSFET) dosimeters] and we validated a MC code for internal breast mammography dosimetry using a homogeneous phantom irradiated by a monoenergetic x‐ray beam. In this study, we perform MC experimental validations for a homogeneous and a 3D‐printed anthropomorphic breast phantom irradiated by a polyenergetic x‐ray spectrum.

## 2 Materials and methods

### 2.A. Mammography system

All measurements were performed using a Mammomat Inspiration (Siemens Healthcare, Forchheim, Germany) digital mammography system. The spectrum selected by the automatic exposure control for the two phantoms evaluated was W/Rh 28 kV. To characterize the x‐ray field and the spectrum to be used in the MC simulations, both the heel effect and the spectrum‐attenuation curve were measured.

A calibrated (certificate no. 17 1861, MEDIX LAB, Versailles, France) ionization chamber (IOC) connected to a dosimeter (Radcal Accu‐Pro model No. 2186, Radcal Corp., Monrovia, CA, USA) was used for all measurements. The chamber consists of two pieces: a converter (model No. 9660) and a dedicated probe for mammography (model No. 10X6‐6M) with an active volume of 6 cm^3^. The attenuation curve and the first half value layer (HVL) of the mammography system x‐ray tube were evaluated without the compression paddle in the field and using a high purity aluminum foil (99.5%). The heel effect was measured by placing the IOC on the support table and recording the air kerma in a grid of 7 (*x* axis, chest wall side) × 5 (*y* axis, chest wall to nipple) designated positions to cover the entire detector area. More information is provided in Fig. S1 of the supporting on‐line material.

### 2.B. Breast phantoms

**Figure 1:**
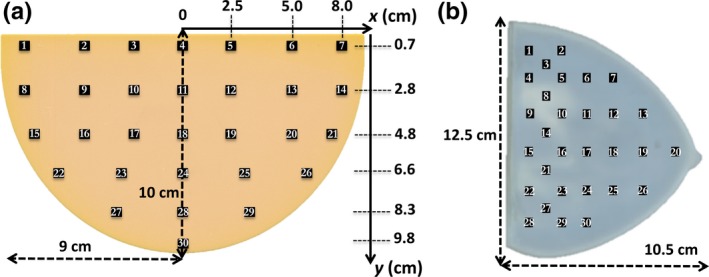
Dosimeter placement on the *xy* plane of the: (a) homogeneous breast phantom; (b) anthropomorphic breast phantom. Drawings are not to scale. [Color figure can be viewed at http://wileyonlinelibrary.com]

A homogeneous semicylindrical phantom (nontarget‐containing slabs of the Model 082, CIRS Inc., Norfolk, Virginia, USA) consisting of a set of 1 cm thick slabs equivalent to 50% glandular/50% adipose breast tissue [Fig. 1(a)] and an anthropomorphic breast phantom [Fig. 1(b)] were used in the measurements.

The anthropomorphic phantom (4.5 cm thick) consists of two 3D printed breast‐shaped sections constructed from real breast CT patient data. Briefly, a patient breast image was automatically classified into voxels representing glandular, adipose, and skin tissue,^19^ and the resulting trinary image underwent simulated mechanical breast compression.^20^ The resulting simulated compressed breast image was 3D printed, resulting in both a digital and a physical anthropomorphic breast phantom with the same tissue distribution. To allow for investigations that required the inclusion of items inside it, as in this study, the phantom was 3D printed in two horizontal pieces, each 2.25 cm thick. The attenuation difference between the glandular and adipose tissue‐equivalent 3D printer materials is 3.5% of that of actual patient tissue. The overall glandularity of the anthropomorphic phantom is 10.9%. Additional details regarding the physical phantom process are reported in the work of Balta et al.^21^

### 2.C. Dosimeter calibration

**Figure 2:**
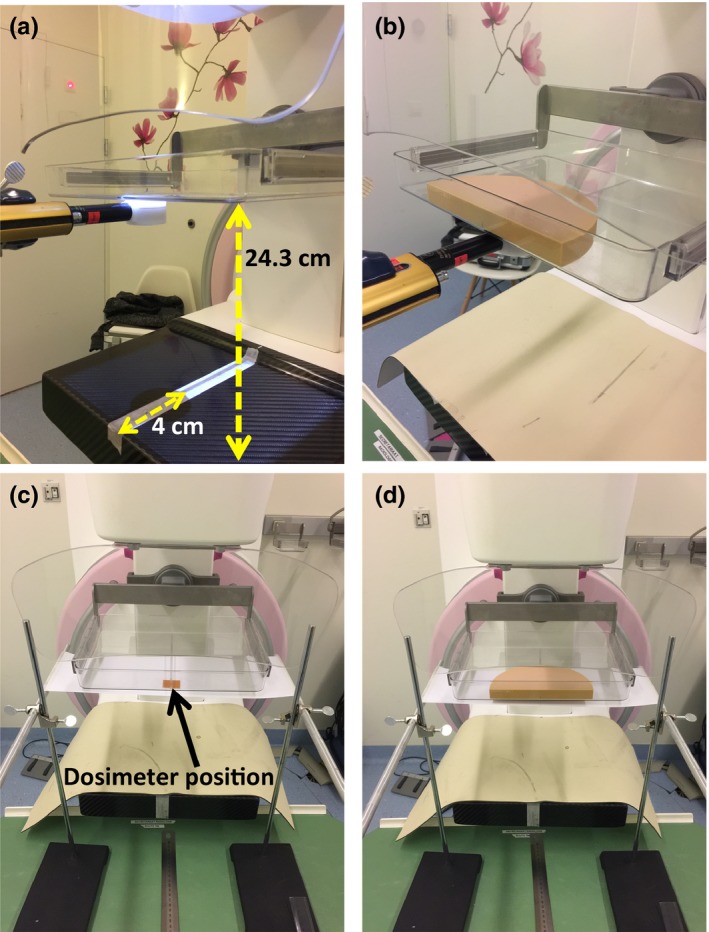
Photos of the calibration setup. (a) The ionization chamber was positioned below the compression paddle, laterally centered and 4 cm from the detector border. (b) Phantom slabs were used to investigate the beam hardening effect. (c) The dosimeters were positioned in the same ionization chamber position. (d) Phantom slabs were also used to investigate the beam hardening effect on dosimeters. [Color figure can be viewed at http://wileyonlinelibrary.com]

All dosimeters were calibrated in terms of air kerma against the calibrated IOCs according to the setup shown in Fig. 2.

During dosimeter calibration, to maximize the available fluence, the compression paddle was positioned as close as possible to the x‐ray tube output port (i.e., 24.3 cm from the breast support paddle) and the IOC was positioned below it [Figs. 2(a) and 2(b)]. Due to the beam hardening effect and the dosimeter's energy dependence,^18^ calibration has to be performed at each investigated depth. Thus, a varying number of phantom slabs were positioned on top of the compression paddle (Fig. 2) to replicate the investigated depths and therefore the expected beam hardening during measurement. The methodologies proposed in Fedon et al.^18^ were followed and several tube current‐exposure time products were manually selected to obtain different air kerma values.

### 2.D. Dosimeter preparation

In our previous study,^18^ we described in detail the experimental procedures, for both calibration and measurement, used for GafChromic™, MOSFET, and TLDs. Here, we briefly summarize the main steps for each dosimeter.

#### 2.D.1. GafChromic™ films

XR‐QA2 GafChromic™ (Ashland, NJ, USA) films have a dose‐sensitive layer specifically designed for low x‐ray energies, via inclusion of high Z element such as Bi, suitable for mammography applications. GafChromic™ films require digitization in a reflective modality in order to evaluate the changes in optical reflectance. An Epson Perfection V750 flat bed scanner was used in reflectance mode to scan the films. The image resolution was set to 72 dpi and images were saved in tagged image file format in 48‐bit RGB mode.

Prior to any scanning session, five blank scans were made in order to warm up the scanner. To better homogenize the pressure over the scanner, a PMMA slab (21 × 30 × 2 cm^3^) was placed on the film during each scan. A single lot of XR‐QA2 films was used throughout this study.

Analysis of the GafChromic™ film was performed using open source software (ImageJ; National Institutes of Health, Bethesda, MD, USA) following the procedure reported in Fedon et al.^18^ which is based on the method proposed by Tomic et al.^22^, ^23^

The XR‐QA2 films were calibrated in terms of net reflectance change (net∆R) versus the air kerma measured at the plane where the films were positioned. Seven different air kerma values were used to obtain a calibration curve in the dose range 1–10 mGy and fitted using a logarithmic function $\left(y=a+\frac{\mathrm{bx}}{\ln x}\right)$. The precision of the calibration functions was tested following the procedure proposed by Devic et al.^24^ where the overall dose uncertainty consists of two terms: the experimental uncertainties (e.g., measurement reproducibility, scan reproducibility, film nonuniformity, etc.) and the uncertainty due to the fitting process (e.g., uncertainty on the fit parameters).

The 2D dose map (in mGy) was obtained using the best calibration fit function and the combined standard uncertainty (k = 1) was estimated as follows:^18^

(1) $$u_{\mathrm{GAF}}=\sqrt{u_{\mathrm{ROI}}^{2}+u_{\mathrm{Calib}}^{2}+u_{\mathrm{IOC}}^{2}}$$

where $u_{\mathrm{ROI}}^{2}$ is a Type A uncertainty for a 1 cm^2^ region of interest (ROI) while $u_{\mathrm{Calib}}^{2}$ and $u_{\mathrm{IOC}}^{2}$ are Type B uncertainties for the calibration and IOC, respectively, estimated on a rectangular‐based distribution.

#### 2.D.2. MOSFET dosimeters

Five high‐sensitivity MOSFET dosimeters, model TN‐1002RD (Best Medical Canada Ltd., Ottawa, Canada) were used in this work in conjunction with the Patient Dose Verification System (model No. TN‐RD‐16) with the high‐sensitivity bias supply setting.

The signal response (∆*V*) of each MOSFET was determined by the difference between the pre‐ and postexposure voltages. Calibration factors (CF) were obtained exposing the dosimeters to a known air kerma value. The final dose value ($\bar{D_{\mathrm{MOSFET}}}$) was obtained by averaging three exposures among the ratio (∆*V*/CF). The combined standard uncertainty (k = 1) for $\bar{D_{\mathrm{MOSFET}}}$ is expressed as follows:^18^

(2) $$u_{\bar{D_{\mathrm{MOSFET}}}}=\sqrt{u_{\Delta V}^{2}+u_{\mathrm{CF}}^{2}+u_{\mathrm{IOC}}^{2}+u_{\mathrm{MOSFET}}^{2}},$$

where $u_{\Delta V}^{2}$ and $u_{\mathrm{CF}}^{2}$ are Type A uncertainties for the signal response and calibration factor, respectively [see Eq. (7)]; and $u_{\mathrm{IOC}}^{2}$ and $u_{\mathrm{MOSFET}}^{2}$ are Type B uncertainties for the IOC and MOSFET accuracy, respectively, all estimated on a rectangular‐based distribution.

#### 2.D.3. Thermoluminescent dosimeters (TLDs)

High sensitivity lithium fluoride (LiF: Mg, Cu, P) TLD chips (TLD‐100H, ThermoFisher Scientific, Waltham, MA, USA) were used in this study. The annealing and reading procedures are described in Fedon et al.^18^ The dose is provided by the following equation:

(3) $$D_{i}^{\mathrm{TLD}}=Q_{i}\;x\;\frac{K_{\mathrm{calib}}}{S_{i}}$$

where *Q*_*i*_ is the *i*th TLD reading (in nC), *S*_*i*_ is a dimensionless sensitivity factor specific for each TLD, and *K*_*calib*_ is the calibration factor (in mGy/nC). No correction was made for the TLD self‐absorption since, at this energy, the TLD thickness was assumed not to attenuate the beam to a significant degree.^25^

At each phantom position (Fig. 1), a final mean dose value ($\bar{D_{\mathrm{TLD}}}$) was calculated by averaging over three TLD values [i.e., $D_{i}^{\mathrm{TLD}}$ in Eq. (3)]. The combined standard uncertainty (k = 1) was estimated as follows:

(4) $$u_{\bar{D_{\mathrm{TLD}}}}=\sqrt{u_{Q}^{2}+u_{S}^{2}+u_{K_{\mathrm{calib}}}^{2}+u_{\mathrm{IOC}}^{2}+u_{TLD-reader}^{2}}$$

where $u_{Q}^{2}$, $u_{S}^{2}$ and $u_{K_{\mathrm{calib}}}^{2}$ are Type A uncertainties for the reading, sensitive factor, and calibration factor, respectively, while $u_{\mathrm{IOC}}^{2}$ and $u_{TLD-reader}^{2}$ are Type B uncertainties for the IOC and TLD‐reader accuracy, respectively, again estimated on a rectangular‐based distribution.

### 2.E. Dose measurements

Thirty fixed positions were selected on the *xy* plane of both phantoms in order to evaluate the dose distribution using the point dosimeters (TLDs and MOSFETs) (Fig. 1), while GafChromic™ films inherently result in the acquisition of continuous 2D dose map distributions within the phantom.

However, in the case of the homogeneous phantom, due to the limited size of the uniform response area of the scanner,^18^ two pieces of film have to be used to cover the entire phantom area. The two GafChromic™ pieces for the homogeneous phantom were separately irradiated and scanned and the two resulting images were fused end‐to‐end using a developed MATLAB code (The MathWorks, Natick, MA, USA). This process was not necessary for the heterogeneous phantom since a single GafChromic™ piece, not larger than the uniform response scanner area, covers the whole anthropomorphic phantom.

With all three dosimeter technologies, dose distributions were investigated at five different depths for the homogeneous phantom while for the anthropomorphic phantom only one depth was accessible (at about 2.25 cm depth).

In the case of TLD and MOSFET dosimeters, for each depth, the dosimeters were placed in the fixed positions depicted in Fig. 1. The measurement was then repeated three times in order to average the final values. In the case of GafChromic™ films a single acquisition with no averaging was performed.

The tube‐current exposure time product selected for the measurements in both phantoms was 360 mAs to ensure an adequate signal at the dose detectors.

### 2.F. Geant4 Monte Carlo simulations

A previously developed MC code^10^, ^15^, ^18^ based on the Geant4 toolkit^26^ (release 10.03, December 2016) was modified to estimate the local dose within the breast phantoms.

The breast phantoms were implemented in the simulation as voxelized volumes with each voxel having a dimension of 0.273 mm^3^. In the case of the homogeneous phantom, all voxels were defined as representing the 50% glandular/50% adipose composition of the CIRS phantom as specified by Byng et al.*,* 27 while for the heterogeneous phantom, the chemical composition for the glandular and adipose tissue‐equivalent 3D printer materials obtained by chemical analysis was used.^21^

To optimize the MC simulation performance in the case of a voxelized geometry, the navigation method (i.e., the method to determine which voxel a particle leaves and enters) *G4VNestedParametrization* was used, as suggested by Schümann et al.^28^ It has been shown that in the case of a heterogeneous voxel geometry this method is about 3% faster than all other methods.

Photoelectric interactions, coherent and incoherent scattering were implemented in the MC code^29^ using the EPDL97 library^30^ by using the Geant4 electromagnetic Physics List Option 4.* The default cut range for photons was used (1 mm, corresponding to an energy of 2.65 keV in 50% glandular breast tissue).

In order to replicate the dosimeter placement in Fig. 1, 30 sensitive volumes were implemented reproducing the TLD characteristics (i.e., chips with dimensions of 3.2 × 3.2 × 0.38 mm^3^, density of 2.48 g/cm^3^, and relative chemical composition of 99.5% LiF, 0.2% Mg, 0.004% Cu, and 0.296% P). The dose evaluated in each sensitive volume (*D*_MC_) was tallied and then converted to dose in air, according to the formula

(5) $$D_{\mathrm{Air}}=D_{\mathrm{MC}}\frac{\bar{{\left(\frac{\mu_{\mathrm{en}}}{\rho }\right)}_{\mathrm{Air}}}}{\bar{{\left(\frac{\mu_{\mathrm{en}}}{\rho }\right)}_{\mathrm{TLD}}}}$$

where $\frac{\bar{{\left(\frac{\mu_{\mathrm{en}}}{\rho }\right)}_{\mathrm{Air}}}}{\bar{{\left(\frac{\mu_{\mathrm{en}}}{\rho }\right)}_{\mathrm{TLD}}}}$ is the ratio of the mass energy‐absorption coefficients for the spectrum for dry air and the TLD material, respectively, both evaluated according to the NIST database.^31^ This ratio is obtained by modifying the original input spectrum taking into account the beam hardening effect in the phantom according to:

(6) $$\bar{{\left(\frac{\mu_{\mathrm{en}}}{\rho }\right)}_{Air/TLD}}=\frac{1}{\psi }\overset{}{\int }{\left(\frac{\mu_{\mathrm{en}}}{\rho }\right)}_{Air/TLD}^{E}\psi_{E}dE$$

where ${\left(\frac{\mu_{\mathrm{en}}}{\rho }\right)}_{Air/TLD}^{E}$ is the mass energy‐absorption coefficients for dry air or TLD material as a function of energy *E, ψ*_*E*_ is the differential energy fluence^32^ while *ψ* is the integral energy fluence.

**Figure 3:**
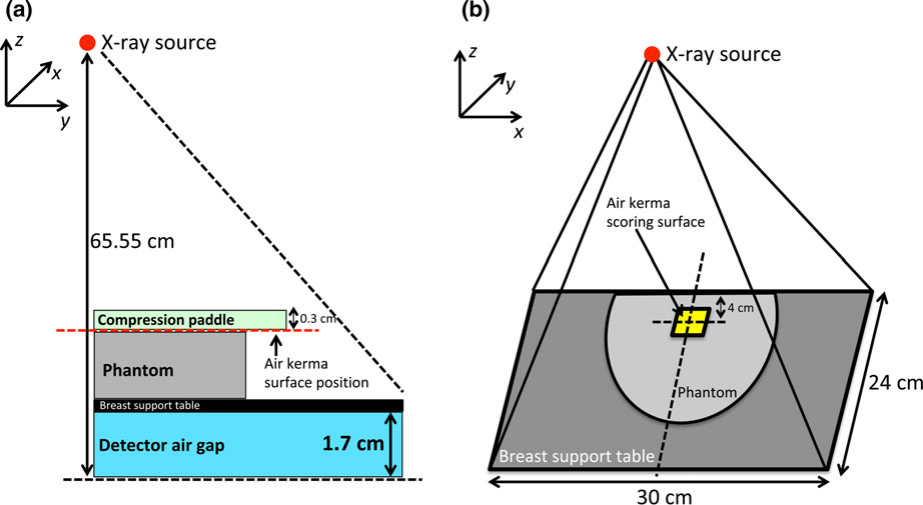
Irradiation geometry implemented in the simulations: (a) lateral view and (b) perspective view. The isotropic x‐ray point source is located 65.55 cm from the detector and the beam is collimated in order to irradiate the entire detector surface. In (a) the red‐dotted line indicates the vertical position of the incident air kerma scoring surface (3 × 3 cm^2^), while in (b) the surface is depicted without the compression paddle for clarity. Drawings are not to scale. [Color figure can be viewed at http://wileyonlinelibrary.com]

The simulated irradiation geometry is shown in Fig. 3. X rays were emitted by an isotropic source collimated to irradiate only the detector surface (30 × 24 cm^2^). The x‐ray source was located 65.55 cm from the detector. The heel effect was included to better reproduce the experimental conditions. For this, the distribution of air kerma measured throughout the support table was used to obtain a surface fit using the commercial software TableCurve 2D (Systat Software Inc., Chicago, IL, USA and SPSS Statistic 20.0, International Business Machines Corp., Armonk, NY, USA) and implemented into the MC simulation to modulate the photon emission.

The compression paddle and the breast support table were implemented in all simulations as a 0.3 cm thick layer of polyethylene terephthalate and a 0.17 cm thick layer of carbon fiber, respectively (data obtained directly from the vendor via personal communication for the purpose of this work).

For each simulation, 2 × 10^10^ photons were simulated to obtain a statistical uncertainty, estimated using the method proposed by Sempau et al.,^33^ below 1% for the dose at the lowest depth (i.e., after 4 cm of phantom material). The simulation time (10 parallel runs of 2 × 10^9^ photons) was on the order of 240 CPU‐hours (on a 3.0 GHz Intel Xeon CPU E5‐2690 v2 computer).

A 2D dose map was obtained by simulating a layer of dimensions 12 × 20 × 0.038 cm^3^ of TLD material.

To normalize the photon fluence in the MC simulation to that used in the experiments, a scale factor was used, defined as the ratio between the experimentally used air kerma (measured by the IOC) and the simulated incident air kerma (analytically evaluated in the MC code) in a square region of area 3 × 3 cm^2^ placed 4 cm from the chest wall, laterally centered and under the compression paddle, as suggested by Sarno et al.^34^

The W/Rh spectrum at 28 kV was modeled using the TASMICS model^35^ by adjusting the thickness of the modeled rhodium filter to minimize the difference between the predicted and the measured attenuation of the seven different aluminum layers previously obtained.

#### 2.F.1. Homogeneous and heterogeneous dose comparison

The above‐described MC code was used to evaluate and compare the average glandular dose (AGD) between a homogeneous and a heterogeneous breast model. The AGD was evaluated following the approach described by Sechopoulos et al.^15^ Specifically, in the case of the heterogeneous breast model, the dose was tallied only in voxels marked as representing glandular tissue; while for the case of the homogeneous breast model, the voxels representing the adipose or glandular tissue were replaced with voxels representing a homogeneous mixture of these two materials, with the mixture fraction corresponding to the overall glandular fraction (by mass) of the anthropomorphic phantom (i.e., 10.9%).

For both simulations, 10^8^ x rays were simulated as emitted by a mammographic system with the characteristics described in the previous section. The number of x rays was enough to obtain an uncertainty level of the total energy below 1%, estimated using the algorithm described by Sempau et al.^33^

## 3 Results

**Figure 4:**
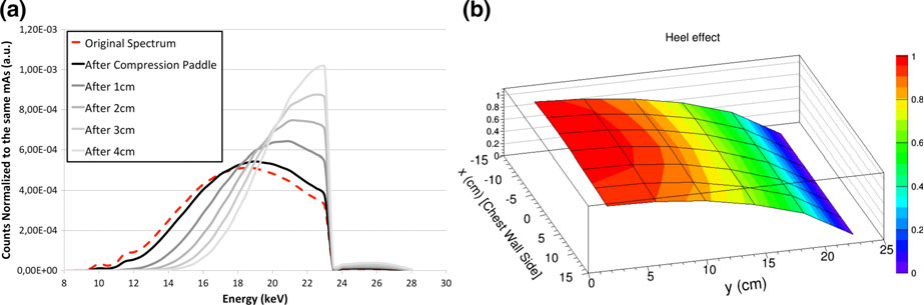
(a) X‐ray spectrum at each homogeneous phantom depth, clearly showing the beam hardening effect. The Monte Carlo input spectrum is shown using the red dashed line. (b) Heel effect implemented in the Monte Carlo simulations. [Color figure can be viewed at http://wileyonlinelibrary.com]

The first HVL for the W/Rh spectrum at 28 kV was 0.55 mm Al. Figure 4(a) shows the beam hardening of the x‐ray beam while traveling through the homogeneous phantom while in Fig. 4(b) the heel effect is depicted.

The best‐fit equation for the heel effect (HE) is

(7) $$\text{HE}=a+b\cdot d^{2}+c\cdot d^{4}$$

where *d* is the distance from the chest wall edge for the heel effect in the *y*‐direction and from the centerline in the *x*‐direction; *a, b,* and *c* are the fit parameters. In particular, *a* = 1.0176, *b* = −0.0008, *c* = −4.8146 × 10^−7^ for *y*‐direction (*r* ^2^ = 0.99), and *a* = 0.9971, *b* = −0.0002, *c* = −3.9390 × 10^−7^ for *x*‐direction (*r* ^2^ = 0.81).

### 3.A. Homogeneous breast phantom

Table 1 lists the calibration curves and the uncertainty fit functions for the GafChromic films™, while calibration factors for MOSFET and TLDs are listed in Table 2.

**Table 1:** Calibration and uncertainty functions for GafChromic™ films

Depth (cm)	Calibration function [*y* = *a* + *bx*/ ln (*x*)]		Total uncertainty functions $\left[y=a+b\exp\left(-x/c\right)\right]$		
	a	b	a	b	c
0	0.5676	−440.7870	4.1906	19.0195	2.1823
1	0.7748	−420.6050	4.4444	17.5723	1.9498
2	0.4796	−382.1020	4.0586	19.7632	1.8211
3	0.8338	−351.8470	4.1864	16.5703	2.0034
4	0.6517	−365.2600	3.9923	16.5250	2.0205

**Table 2:** Calibration factors for MOSFET and TLDs, as a function of phantom depth. For MOSFET, calibration factors are specific to each individual dosimeter evaluated; in this study five MOSFET dosimeters were used

Depth (cm)	Range for calibration factor — MOSFET (min–max) mV/mGy	Calibration factor for TLDs mGy/nC
0	(1.64 ± 0.06) — (1.75 ± 0.06)	(1.11 ± 0.01) 10^−2^
1	(1.81 ± 0.06) — (1.95 ± 0.06)	(1.04 ± 0.01)·10^−2^
2	(2.07 ± 0.07) — (2.16 ± 0.08)	(1.03 ± 0.01) 10^−2^
3	(2.46 ± 0.10) — (2.60 ± 0.09)	(0.93 ± 0.01) 10^−2^
4	(2.68 ± 0.10) — (2.82 ± 0.12)	(0.93 ± 0.01) 10^−2^

**Figure 5:**
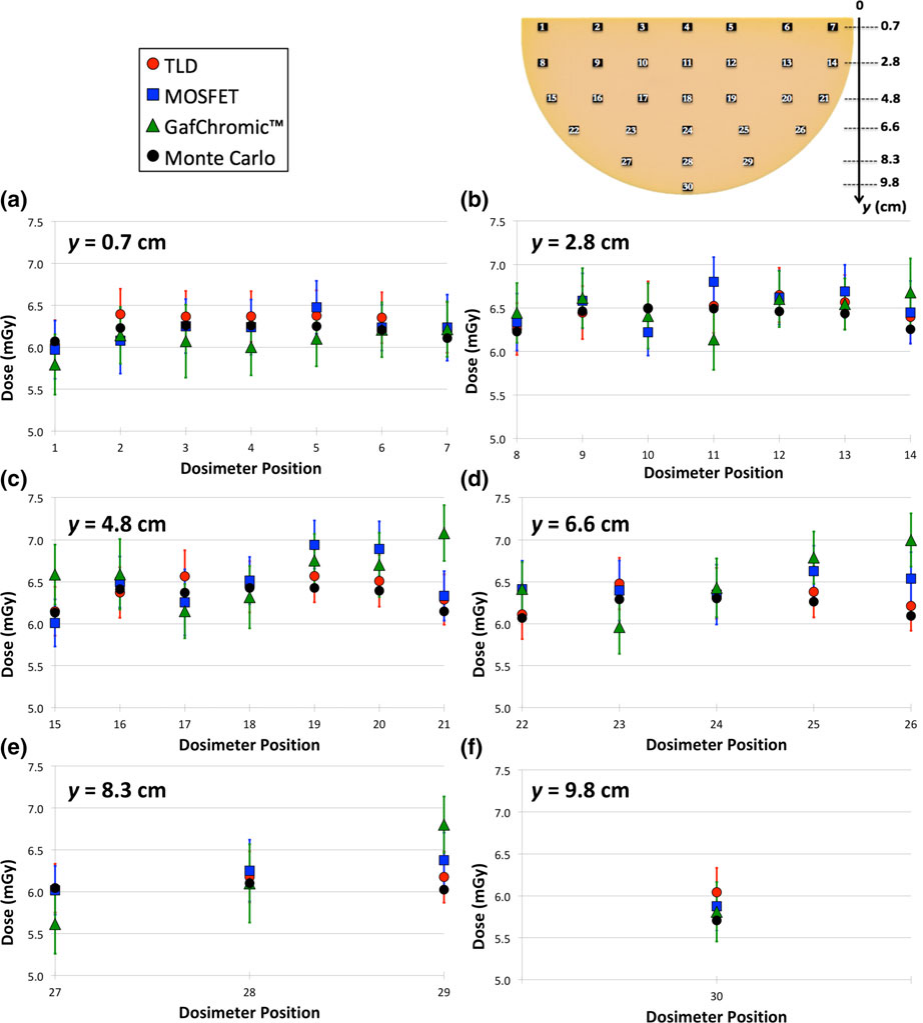
Dose comparison between TLD (red dots), MOSFET (blue squares), GafChromic™ film (green triangles), and Monte Carlo simulations (black dots) at 1‐cm depth of the homogeneous phantom. In all graphs the uncertainty bars refer to the combined standard uncertainty (k = 1) and the dosimeter positions refer to Fig. 1(a). The distance (*y*) from the chest wall is noted in the upper left corner of each graph. [Color figure can be viewed at http://wileyonlinelibrary.com]

The comparison among all experimental measurements and the MC simulation for the depth of 1 cm is shown in Fig. 5. The results for all other phantom depths can be found in Figures S2, S3, S4, and S5 of the supporting on‐line material. A good agreement, within one combined standard uncertainty (k = 1), is found among all experimental data, at all depths.

**Figure 6:**
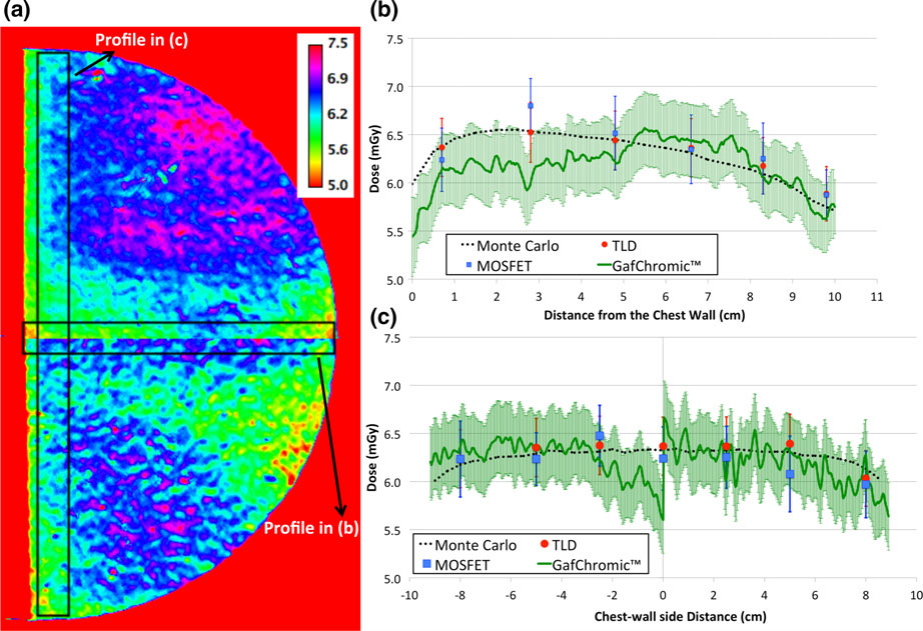
(a) 2D dose map obtained using the GafChromic™ film at the depth of 1 cm for the homogeneous phantom. The values shown in the color legends are in units of mGy. In (b) and (c) the comparison among the experimental data obtained using TLD (red dots), MOSFET (blue squares), GafChromic™ film (green solid line), and the Monte Carlo data (dotted black line) is reported for the chest wall to nipple central profile and along the chest wall side profile, respectively. The uncertainty bars represent the combined standard uncertainty (k = 1). [Color figure can be viewed at http://wileyonlinelibrary.com]

Figure 6(a) show the 2D dose map for GafChromic™ film at the depth of 1 cm. The two pieces of GafChromic™ film used to cover the entire phantom can be easily recognized: the central horizontal line represents the line along which the two pieces were fused, explaining the discontinuity in the results. Figures 6(b) and 6(c) show the two profiles obtained in the ROIs depicted in Fig. 6(a).

In Fig. 6(b), the impact of the heel effect on the dose can be seen in both the MC simulations and the experimental data. The decrease in dose in the ~2 cm closest to the chest wall toward the edge is mainly due to the lower scatter radiation in this area, while from this region toward the nipple the heel effect becomes dominant.

The inhomogeneity visible in the GafChromic™ film (i.e., between the two fused images) can be explained by the inherently nonuniform nature of the films.^18^ However, this inhomogeneity is less evident if the uncertainties on the experimental values are taken into account, as can be seen in Fig. 6(c). As expected, as the phantom depth increases, the percentage uncertainty also increases (Table 3). In particular, for the GafChromic™ film, at the phantom depth of 1 cm, the mean combined uncertainty is 5.5%.

**Table 3:** Range of the combined standard uncertainty (k = 1) for all three dosimeters (TLD, MOSFET, and GafChromic™) as function of depth in the homogeneous phantom

	TLD (%)			MOSFET (%)			GafChromic™ (%)		
	Min	Mean	Max	Min	Mean	Max	Min	Mean	Max
Depth									
0 cm	4.7	4.8	4.9	4.1	4.6	5.4	4.2	5.0	6.4
1 cm	4.7	4.8	4.9	4.1	5.0	6.4	4.5	5.5	7.6
2 cm	4.7	4.9	5.2	4.3	5.2	7.5	6.3	7.2	8.6
3 cm	4.7	5.1	6.0	4.3	6.3	8.6	8.1	9.3	11.7
4 cm	4.7	5.3	7.9	5.1	6.3	9.4	8.5	9.6	12.2

When averaging all 30 dosimeters located at the same depth, an average dose decrease of ~93% is observed between the entrance and the 4 cm deep layer (see Figure S6 on the additional supporting on‐line material).

### 3.B. Anthropomorphic breast phantom

**Figure 7:**
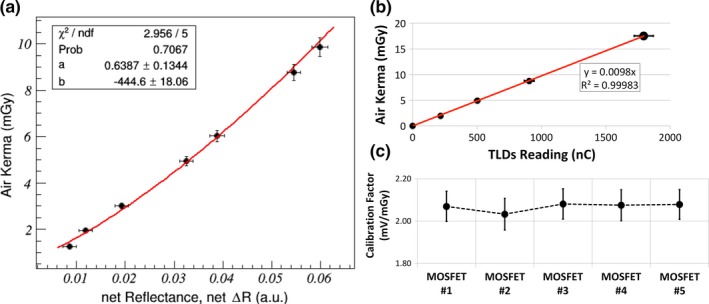
(a) Fit‐calibration curve for the GafChromic™ film. (b) Fit‐calibration line for TLDs. (c) Single calibration values for each MOSFET. [Color figure can be viewed at http://wileyonlinelibrary.com]

The dosimeter calibration when the dosimeters are located in the central layer of the anthropomorphic phantom is shown in Fig. 7.

**Figure 8:**
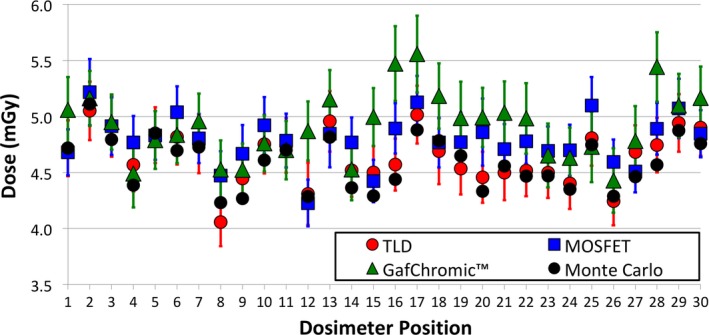
Dose comparison among TLD (red dots), MOSFET (blue squares), GafChromic™ film (green triangles), and Monte Carlo simulations (black dots). In the graphs the uncertainty bars refer to the combined standard uncertainty (k = 1) and the dosimeter positions refer to Fig. 1(b). [Color figure can be viewed at http://wileyonlinelibrary.com]

Figure 8 shows the comparison among all experimental measurements and MC simulations. The results show a good agreement between TLDs, MOSFET, and MC simulations, while for the GafChromic™ a higher deviation was found for some locations (e.g., #15–17 and #28).

The range of experimental uncertainty is reported in Table 4.

**Table 4:** Range of the combined standard uncertainty (k = 1) for all three dosimeters (TLD, MOSFET, and GafChromic™) for the measurements at the center of the anthropomorphic breast phantom

TLD (%)			MOSFET (%)			GafChromic™ (%)		
Min	Mean	Max	Min	Mean	Max	Min	Mean	Max
5.1	5.3	6.4	4.1	4.9	6.2	6.2	6.6	7.1

**Figure 9:**
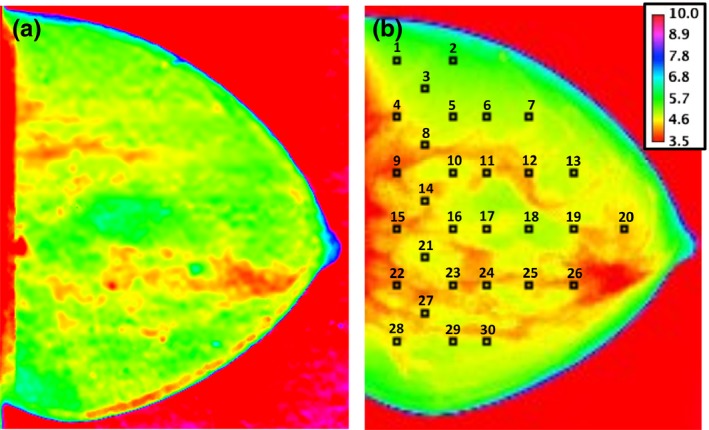
2D dose map obtained using GafChromic™ film (a) and Monte Carlo 2D dose map (b). The values in the legend are in units of mGy and correspond to both maps. In (b) the dosimetry position is also reported. [Color figure can be viewed at http://wileyonlinelibrary.com]

A comparison of the 2D dose map obtained experimentally with GafChromic™ film and that obtained with the MC simulation is shown in Fig. 9. The MC simulation reproduced with good agreement the experimental map, as expected depicting the fibro‐glandular structure of the breast phantom with higher spatial resolution.

**Figure 10:**
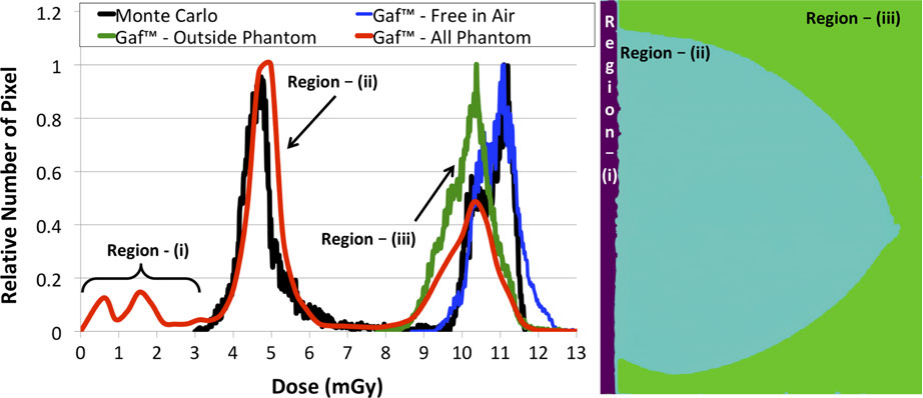
Normalized dose value histogram comparison between Monte Carlo simulation (in black) and GafChromic™ film (red, blue, and green curves) for the anthropomorphic phantom. In red the results for all three regions, (i)‐(ii)‐(iii) are included. For region (iii) the results for when the in‐phantom calibration (green line) and when the free‐in‐air calibration (blue) is used are shown. The histogram thresholds used to segment the three regions (figure on the right side) are shown with dashed vertical lines. [Color figure can be viewed at http://wileyonlinelibrary.com]

Figure 10 shows the dose value histogram of the dose maps shown in Fig. 9, each one normalized separately to its maximum value being unity, in addition to the dose map obtained by a GafChromic™ film when the phantom is removed (i.e., free in air).

Three regions can be distinguished in these histograms, based on their dose values. They are: (a) values lower than 3 mGy, corresponding to the nondirectly irradiated sections of the maps, posterior to the chest wall edge of the x‐ray field (no data were obtained for this area with the MC simulation); (b) values within 3–8 mGy, corresponding to the values within the phantom, where it can be seen that the dose distribution for GafChromic™ is slightly higher than that of the MC simulation; and (3) values higher than 8 mGy, corresponding to the region outside the phantom, where a visible discrepancy between the MC and the GafChromic™ results can be seen. This deviation is due to the fact that for this extra‐phantom region (i.e., free in air) a different calibration curve should be used. For this, the dose value histogram when GafChromic™ film is used free in air, with the phantom removed, and with the calibration data for the 0 cm depth in Table 1 is used. A better agreement is then found.

### 3.C. Homogeneous and heterogeneous dose comparison

For the heterogeneous anthropomorphic breast phantom, the resulting AGD is 0.10 mGy. For the homogeneous breast phantom with the same glandular fraction (i.e., 10.9%) under the same MC conditions, the AGD is 0.11 mGy. This 10% difference is consistent with previous findings,^14^, ^15^, ^16^ due to the glandular tissue being homogeneously distributed throughout the entire breast. Thus, a higher amount of glandular tissue is present in the upper part of the breast, facing the x‐ray output, and therefore receiving more radiation dose. When the real glandular distribution is considered (i.e., using the anthropomorphic phantom), there is less glandular tissue in the upper part of the breast, leading to a lower AGD value.^15^

## 4 Discussion

As anticipated in our previous work,^18^ this study aimed to validate the dose estimation by MC simulations at the local level within and throughout a breast model undergoing irradiation with mammographic conditions. In particular, the MC validation in a 3D‐printed anthropomorphic breast phantom provides insight into the ongoing development of new breast dosimetry methods. The validated MC code can therefore be used to obtain patient‐specific dose estimates, as well as new dose conversion coefficients in heterogeneous, nonuniform breast models, the latter as undertaken by the joint Task Group 282 of the American Association of Physicists in Medicine (AAPM) and the European Federation of Organizations for Medical Physics (EFOMP).^36^

Good agreement was found between all experimental measurements and the MC simulations, mostly within the combined standard uncertainty. Higher discrepancies were observed for the GafChromic film™, which, as with the inhomogeneity visible in Fig. 6(a), are due to the inherent inhomogeneities of this technology^18^ due to issues such as the spreading of the sensitive layer and quantitative variability within the same batch.^37^, ^38^

The dose profiles along the chest wall to nipple direction [Fig. 6(b)] show that the heel effect is detected by all three experimental devices and that it is properly reflected in the MC simulations. The heel effect modulates the dose profile toward the nipple: a different trend is expected if no heel effect is present, as shown in our previous investigation.^18^

As expected, the uncertainty increases as a function of depth due to the increase in the percentage contribution of Type A uncertainties for GafChromic™films [i.e., $u_{\mathrm{ROI}}^{2}$ in Eq. (1)] and for MOSFET [i.e., $u_{\Delta V}^{2}$ and $u_{\mathrm{CF}}^{2}$ in Eq. (2)] when approaching the lower boundary of the detection range.

As shown in Figure S5 (additional supporting on‐line material), the average experimental dose at the depth of 1 cm is about 6.4 mGy. However, a large portion of the breast phantom receives a dose greater than 6.4 mGy [Fig. 6(a)]. This work shows that an average dose decrease of ~93% is observed between the entrance and the 4 cm deep layer, in agreement with the work of Sechopoulos et al.^39^ who found a reduction of 94% in dose after the same thickness of breast phantom.

In the case of the anthropomorphic breast phantom, the comparison between MC data and GafChromic™ film showed higher discrepancies (i.e., outside one combined standard uncertainty) than with the other two technologies (Fig. 8). In particular, in the dosimetry position between the boundary of adipose and glandular tissue (e.g., #12, #16, #17, and #28 in Fig. 8) the measurements agree with the MC data within the 99% confidence interval.

As shown in Fig. 9, the local dose at a single depth can vary by almost a factor of 2 between a glandular and an adipose region. This variation in local dose deposition points to the importance of using a breast model based on real glandular tissue distribution, obtained by 3D breast imaging.

Notwithstanding the abovementioned inherent issues^18^ with GafChromic™ film, the use of this dosimeter technology in heterogeneous phantoms to assess the dose distribution appears feasible (if sufficient entrance air kerma is delivered) and it confirms previous conclusions of the work of Sarno et al.^40^

Finally, the impact of using the incorrect calibration data when measuring dose inside and outside a phantom was shown (Fig. 10). This emphasizes the reason for our initial validation being performed with a monochromatic beam, so as to simplify the process while the methodology for internal breast dosimetry was being developed and optimized.^18^

The main limitation in this study is the range of parameters studied. This work has been performed using only one system, one x‐ray spectrum, and only two phantoms (albeit one homogeneous and one heterogeneous). The use of other systems and spectra could enhance the robustness of the validation, by including different testing conditions in, e.g., imaging geometry, heel effect, anode/filter combinations, etc. However, a much larger variation in conditions that could ever be encountered by using a different digital mammography system has already been tested if the validation performed in our previous work, using mono‐energetic x rays from a synchrotron beamline, is considered together with the study performed here. It should also be noted that the methodology proposed here is not vendor specific and can be easily adjusted to the specifications of different mammography systems; while the MC results can also be extended to other geometries, x‐ray spectra, and phantoms. However, TLD and GafChromic™ measurements are time consuming in terms of reading time (for TLDs) and calibration procedure (for GafChromic™). Additional measurements in terms of depths in the breast, other systems, spectra, and/or phantoms each require a considerable amount of time. In the case of the heterogeneous phantom measurements, the dosimeter calibration was performed in a heterogeneous background (i.e., with the limitations of the dosimeter location). Therefore, the actual background in which each dosimeter was calibrated is a source of uncertainty, which is included in the calibration process itself. Finally, the differences in attenuation between the two tissue‐equivalent materials of the anthropomorphic phantom and actual adipose and glandular breast material are x‐ray energy‐dependent. However, for the spectrum used here, the error in equivalence in attenuation difference between the two materials is 3.5%.

## 5 Conclusions

In this work, we performed experimental validations (using GafChromic™ films, MOSFET, and TLDs) of an MC code at the level of local dose deposition and in absolute terms for a homogeneous and a 3D anthropomorphic heterogeneous breast phantom under mammographic conditions. The results showed a good agreement between experimental measurements and MC data within the experimental uncertainty.

The proposed methodology for validating MC code in a heterogeneous background can be successfully used to calculate patient‐specific dose estimates^37^ with actual patient tissue distributions, potentially obtained with 3D or pseudo‐3D modalities, or new dose conversion coefficients for heterogeneous breast models.

## Conflicts of interest

The authors have no relevant conflicts of interest to disclose.

## Supporting information


**Fig. S1.** Graphical representation of the grid‐like distribution of positions at which the heel effect was measured with an ionization chamber (diameter of 4.4 cm). 35 positions (white circles) were selected on the detector cover (gray background). The dose measured by the ionization chamber was recorded in each position as an average value of three consecutive exposures. The drawing is not to scale.
**Fig. S2.** Dose comparison between TLD (red dots), MOSFET (blue squares), GafChromic™ film (green triangles), and Monte Carlo simulations (black dots) at 0‐cm depth of the homogeneous phantom. In all graphs the uncertainty bars refer to the combined standard uncertainty (k = 1) and the dosimeter positions refer to Fig. 1(a). The distance (*y*) from the chest wall is noted in the upper left corner of each graph.
**Fig. S3.** Dose comparison between TLD (red dots), MOSFET (blue squares), GafChromic™ film (green triangles), and Monte Carlo simulations (black dots) at 2‐cm depth of the homogeneous phantom. In all graphs the uncertainty bars refer to the combined standard uncertainty (k = 1) and the dosimeter positions refer to Fig. 1(a). The distance (*y*) from the chest wall is noted in the upper left corner of each graph.
**Fig. S4.** Dose comparison between TLD (red dots), MOSFET (blue squares), GafChromic™ film (green triangles), and Monte Carlo simulations (black dots) at 3‐cm depth of the homogeneous phantom. In all graphs the uncertainty bars refer to the combined standard uncertainty (k = 1) and the dosimeter positions refer to Fig. 1(a). The distance (*y*) from the chest wall is noted in the upper left corner of each graph.
**Fig. S5.** Dose comparison between TLD (red dots), MOSFET (blue squares), GafChromic™ film (green triangles), and Monte Carlo simulations (black dots) at 4‐cm depth of the homogeneous phantom. In all graphs the uncertainty bars refer to the combined standard uncertainty (k = 1) and the dosimeter positions refer to Fig. 1(a). The distance (*y*) from the chest wall is noted in the upper left corner of each graph.
**Fig. S6.** Mean dose obtained averaging all 30 values as a function of the increasing depth in the homogeneous phantom. The uncertainty bars refer to the combined standard uncertainty (k = 1).

## Note

1
http://geant4.cern.ch/collaboration/working_groups/electromagnetic/physlist10.0.shtml
