# Supplementary material for: Internal breast dosimetry in mammography: Monte Carlo validation in homogeneous and anthropomorphic breast phantoms with a clinical mammography system
Source: Med Phys. 2018 Jul 18;45(8):3950–61. doi: 10.1002/mp.13069 (PMC6099211; doi:10.1002/mp.13069)
Supplement: Supplementary file 1 — Fig. S1. Graphical representation of the grid‐like distribution of positions at which the heel effect was measured with an ionization chamber (diameter of 4.4 cm). 35 positions (white circles) were selected on the detector cover (gray background). The dose measured by the ionization chamber was recorded in each position as an average value of three consecutive exposures. The drawing is not to scale. Fig. S2. Dose comparison between TLD (red dots), MOSFET (blue squares), GafChromic™ film (green triangles), and Monte Carlo simulations (black dots) at 0‐cm depth of the homogeneous phantom. In all graphs the uncertainty bars refer to the combined standard uncertainty (k = 1) and the dosimeter positions refer to Fig. 1(a). The distance (y) from the chest wall is noted in the upper left corner of each graph. Fig. S3. Dose comparison between TLD (red dots), MOSFET (blue squares), GafChromic™ film (green triangles), and Monte Carlo simulations (black dots) at 2‐cm depth of the homogeneous phantom. In all graphs the uncertainty bars refer to the combined standard uncertainty (k = 1) and the dosimeter positions refer to Fig. 1(a). The distance (y) from the chest wall is noted in the upper left corner of each graph. Fig. S4. Dose comparison between TLD (red dots), MOSFET (blue squares), GafChromic™ film (green triangles), and Monte Carlo simulations (black dots) at 3‐cm depth of the homogeneous phantom. In all graphs the uncertainty bars refer to the combined standard uncertainty (k = 1) and the dosimeter positions refer to Fig. 1(a). The distance (y) from the chest wall is noted in the upper left corner of each graph. Fig. S5. Dose comparison between TLD (red dots), MOSFET (blue squares), GafChromic™ film (green triangles), and Monte Carlo simulations (black dots) at 4‐cm depth of the homogeneous phantom. In all graphs the uncertainty bars refer to the combined standard uncertainty (k = 1) and the dosimeter positions refer to Fig. 1(a). The distance (y) from the chest wall is [file MP-45-3950-s001.pdf]

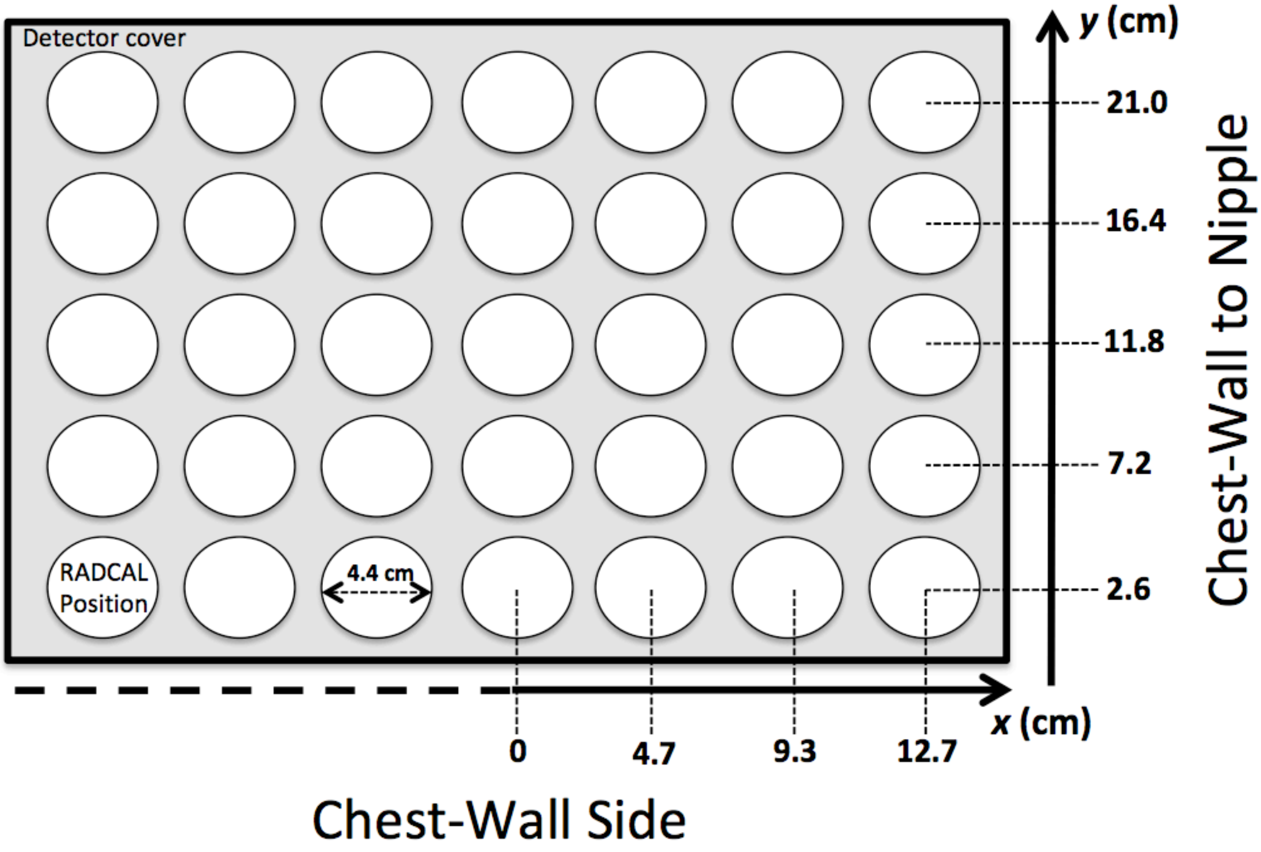

Fig. S1. Graphical representation of the grid-like distribution of positions at which the heel effect was measured with an ionization chamber (diameter of 4.4 cm). 35 positions (white circles) were selected on the detector cover (gray background). The dose measured by the ionization chamber was recorded in each position as an average value of three consecutive exposures. The drawing is not to scale.

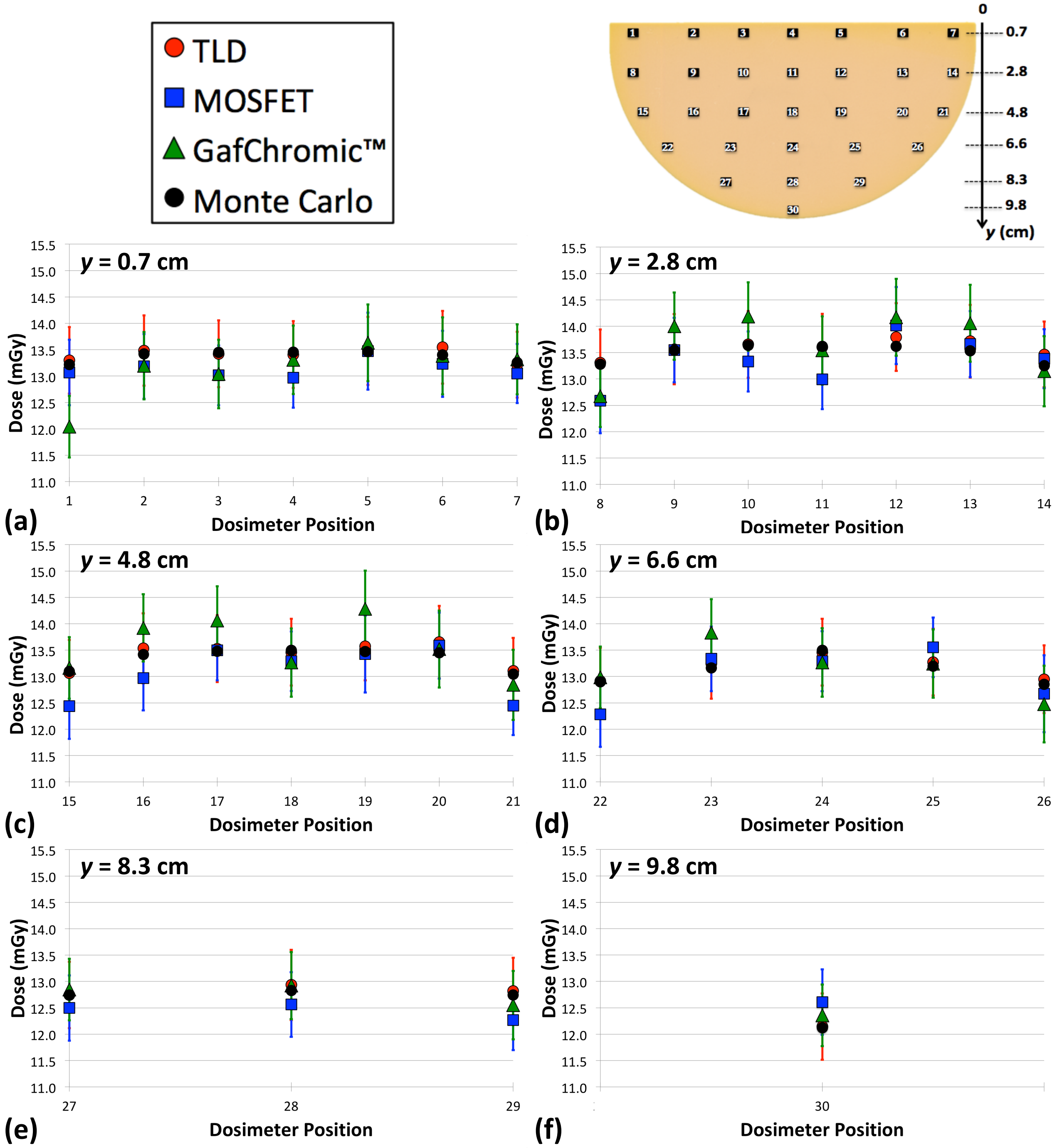

Fig. S2. Dose comparison between TLD (red dots), MOSFET (blue squares), GafChromic™ film (green triangles) and Monte Carlo simulations (black dots) at 0-cm depth of the homogeneous phantom. In all graphs the uncertainty bars refer to the combined standard uncertainty (k=1) and the dosimeter positions refer to Figure (1a). The distance (*y*) from the chest wall is noted in the upper left corner of each graph.

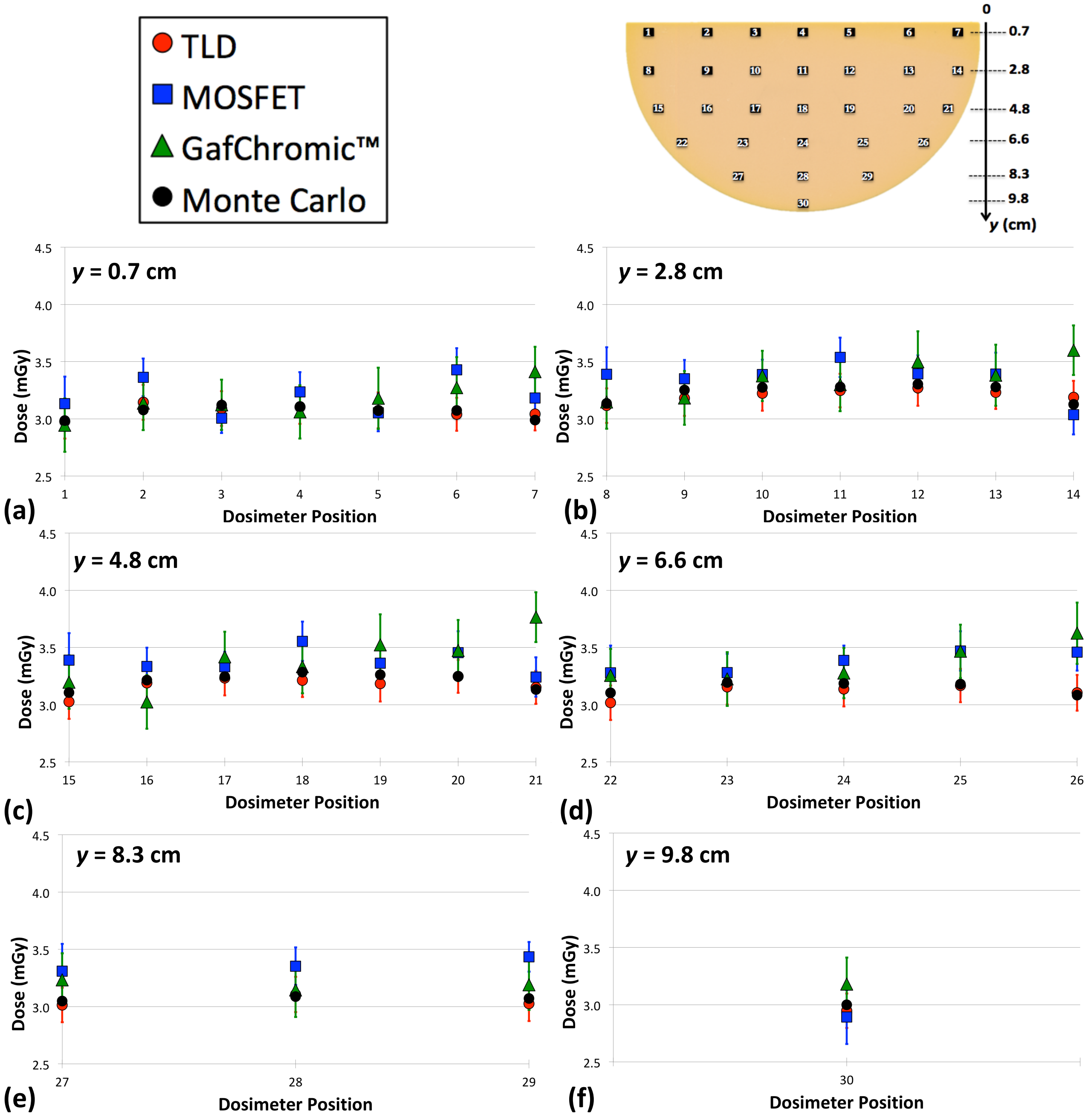

Fig. S3. Dose comparison between TLD (red dots), MOSFET (blue squares), GafChromic™ film (green triangles) and Monte Carlo simulations (black dots) at 2-cm depth of the homogeneous phantom. In all graphs the uncertainty bars refer to the combined standard uncertainty (k=1) and the dosimeter positions refer to Figure (1a). The distance (*y*) from the chest wall is noted in the upper left corner of each graph..

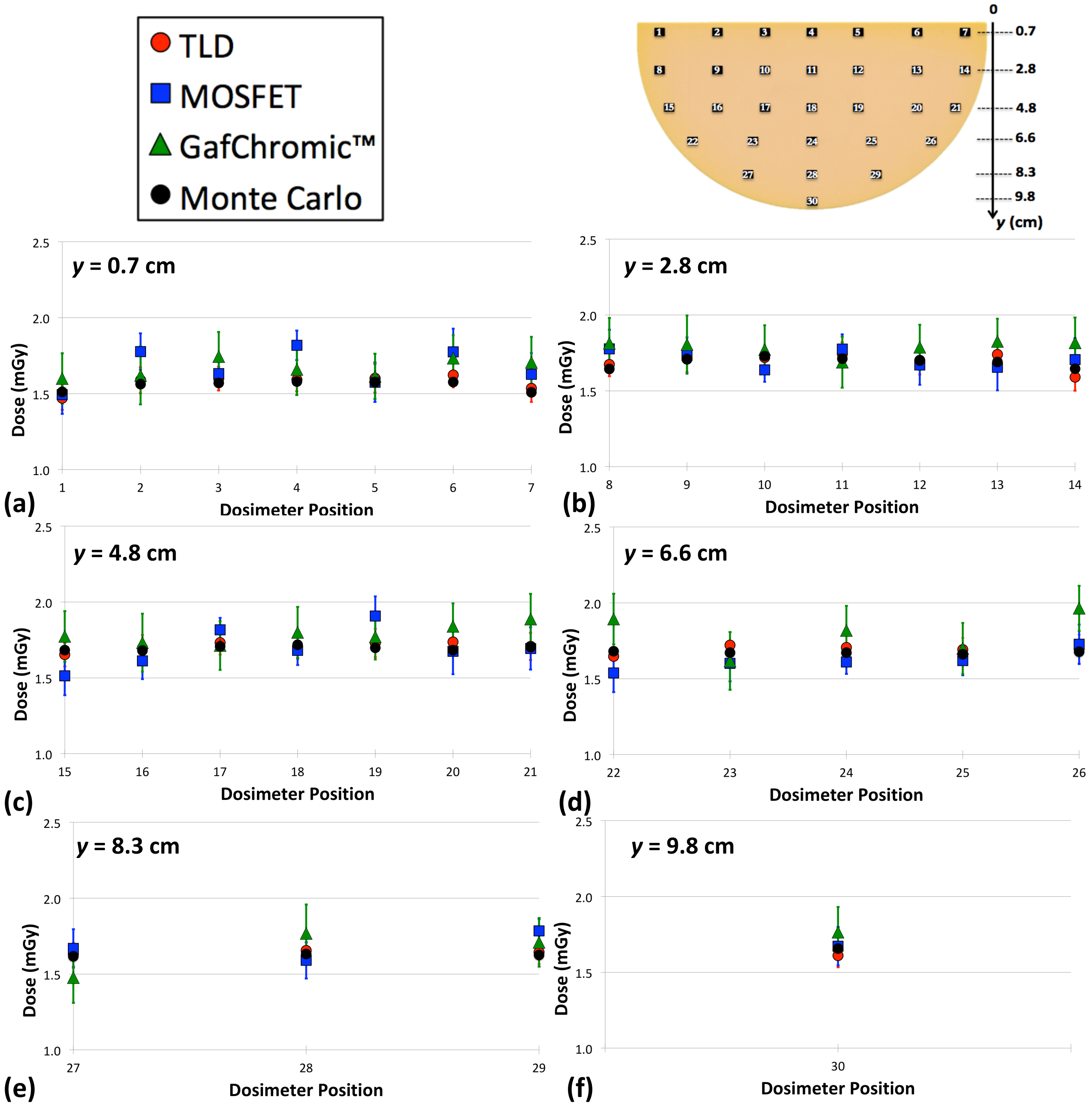

Fig. S4. Dose comparison between TLD (red dots), MOSFET (blue squares), GafChromic™ film (green triangles) and Monte Carlo simulations (black dots) at 3-cm depth of the homogeneous phantom. In all graphs the uncertainty bars refer to the combined standard uncertainty (k=1) and the dosimeter positions refer to Figure (1a). The distance (*y*) from the chest wall is noted in the upper left corner of each graph.

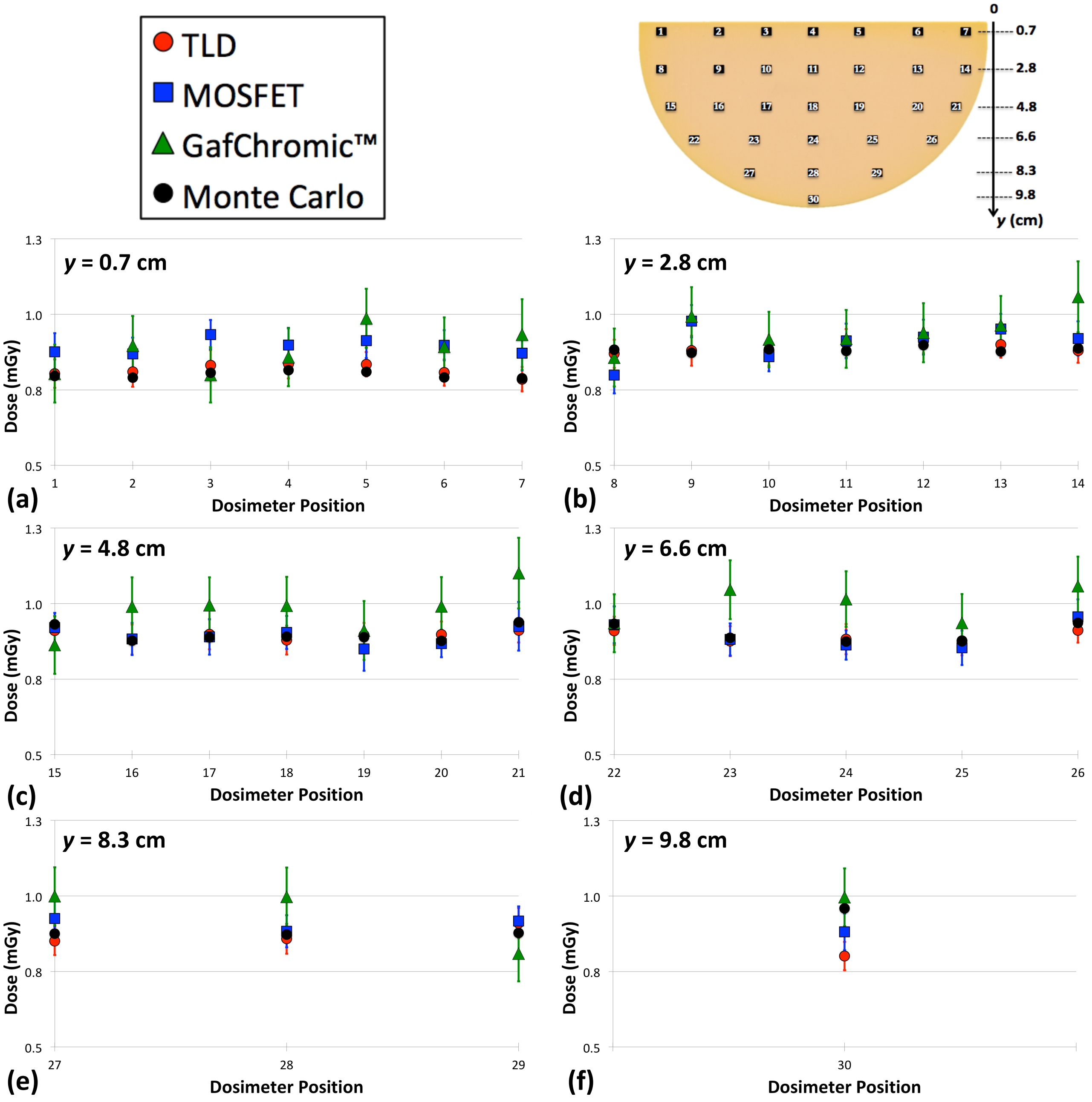

Fig. S5. Dose comparison between TLD (red dots), MOSFET (blue squares), GafChromic™ film (green triangles) and Monte Carlo simulations (black dots) at 4-cm depth of the homogeneous phantom. In all graphs the uncertainty bars refer to the combined standard uncertainty (k=1) and the dosimeter positions refer to Figure (1a). The distance (y) from the chest wall is noted in the upper left corner of each graph.

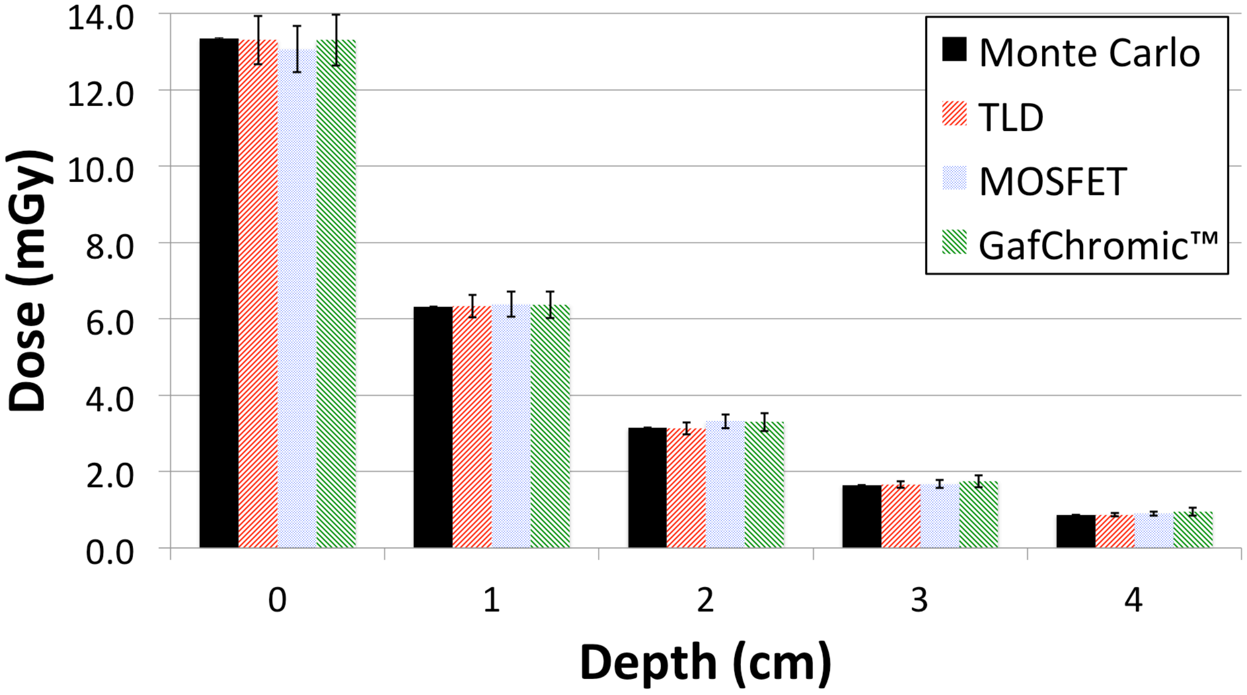

Fig. S6. Mean dose obtained averaging all thirty values as a function of the increasing depth in the homogeneous phantom. The uncertainty bars refer to the combined standard uncertainty (k=1).
